# Enhancing public health through multi-stakeholder collaboration in Africa

**DOI:** 10.1097/MS9.0000000000002532

**Published:** 2024-09-05

**Authors:** Chimwemwe Ngoma, William K. B. Phiri, Robert Chidzaye, Sahan Lungu, Apatsa Matatiyo, Martha Shantel Mwase, Wanangwa Nyimba

**Affiliations:** a Tobacco Harm Reduction Scholarship Programme, Knowledge Action Change, London, UK; b Department of Research and Innovation, ThinkSmart Consulting; c Department of Social Work, University of Lilongwe; d Department of Research and Innovation, Green Chairs Consulting, Lilongwe; e Ntchisi District Hospital, Ministry of Health; f University of North Carolina Cancer Research; g Department of Information & Communication Technology, Mzuzu University; h Department of Business and Communication Studies, University of Livingstonia, Mzuzu, Malawi

## Introduction

Public health policies are crucial in safeguarding the well-being of communities, particularly in low and middle-income regions like Africa, where social, political, environmental, and economic conditions create numerous health vulnerabilities^1,2^. The challenges range from infectious diseases like Ebola virus disease, yellow fever, tuberculosis, malaria, and cholera to non-communicable diseases such as cardiovascular diseases, respiratory disorders, and cancers, which are often exacerbated by high-risk behaviors like tobacco use^1,3^.

Developing and implementing effective public health interventions require an understanding of these issues and collaborative efforts that go beyond traditional boundaries^4^. While there are established frameworks for stakeholder engagement, there still remains little progress for multi-sector collaboration in most African countries^5^. For instance, in some African countries, critical and interrelated sectors like nutrition, tobacco control, and other public health issues are regulated by separate ministries like health, trade, and treasury^6^. This disjointed approach hinders the potential synergies that could be achieved by coordinating efforts and aligning policies around common public health goals.

This paper explores the dynamics of multi-stakeholder collaboration with the aim of understanding how diverse stakeholders in Africa can effectively work together to develop evidence-driven and contextually relevant public health interventions. It provides insights into the challenges, opportunities, and best practices surrounding collaborative efforts in African public health.

## Stakeholder contributions

Having established the importance of multi-stakeholder collaboration in addressing public health challenges, we now look at the various stakeholders involved. Stakeholders across diverse sectors contribute to the development and successful implementation of public health interventions that meet new priorities and evolving expectations^7,8^. This approach is important in Africa as the continent is characterized by health disparities and emerging diseases^9^. Stakeholder Theory, a key theoretical framework in understanding collaborative governance, emphasizes the importance of considering the interests and inputs of all stakeholders involved^10^. In public health, stakeholders represent a wide range of actors, including but not limited to researchers, healthcare professionals, policymakers, industry leaders, community representatives, advocacy groups, and the citizen patient.

Researchers provide evidence-based insights and recommendations that serve as the foundation for informed policy decisions^11^. Leaders from the healthcare sector, pharmaceutical companies, and related industries also bring to the table resources, technical knowledge, and industry insights that contribute to the design of effective and science-based strategies^12^.

The perspectives and experiences of citizen patients provide valuable insights often overlooked by traditional stakeholders. Additionally, recognizing the wisdom and voices of grassroots organizations, community leaders, advocacy groups, and marginalized individuals is key to crafting comprehensive and inclusive public health policies^13^. This approach aligns with the Alma-Ata principle of community participation in healthcare decision-making, which emphasizes the importance of involving communities and individuals in shaping their healthcare priorities and solutions^14^.

## Success stories achieved through multi-stakeholder collaboration

To illustrate the practical impact of stakeholder contributions, we will now explore several success stories where multi-stakeholder collaboration has led to significant public health achievements. These case studies provide concrete examples of how diverse actors can work together to address complex health issues.

## Combating HIV/AIDS

The collective efforts to combat the transmission of HIV/AIDS provide a compelling illustration of successful multi-stakeholder collaboration. This collaborative initiative encompasses a diverse range of stakeholders who have united to promote comprehensive HIV prevention strategies, including the adoption of new technologies such as pre-exposure prophylaxis (PrEP) and post- exposure prophylaxis (PEP), alongside condom use and traditional interventions such as abstinence and remaining faithful to one sexual partner^15,16^.

The impact of these collaborative efforts is underscored by positive outcomes in the reduction of HIV prevalence rates and the enhancement of overall well-being within affected communities^17^, for instance, new HIV infections have dropped by 39% since 2010, from 2.1 million to 1.3 million in 2023. Additionally, between 2004 and 2010, the number of AIDS-related deaths dropped by 69%. Since 2010, this number has further declined by 51%, from 1.3 million in 2010 to 630 000 in 2023 (Table 1). Furthermore, as of 2023, 86% of all HIV-positive individuals are aware of their status, 89% are receiving treatment, and 93% have viral suppression^18^.

**Table 1:** Trends in HIV infections, deaths, treatment and resources (2000–2023)

Year	New HIV infections (millions)	AIDS-related deaths	People accessing antiretroviral therapy (%)	Resource availability (US$)
2000	2.8	1.8 million	1.8	5.1 billion
2005	2.5	2 million	6.4	9.3 billion
2010	2.1	1.3 million	24.0	16.7 billion
2020	1.5	730 000	67.7	21.5 billion
2022	1.4	670 000	74.2	20.8 billion
2023	1.3	630 000	76.9	19.8 billion

Adapted from UNAIDS (2024). Global HIV & AIDS statistics – *Fact sheet*. https://www.unaids.org/en/resources/fact-sheet

The success is a result of collective action by various actors. Pharmaceutical companies ensured the production and distribution of prevention tools^19^. Health professionals and NGOs provided accurate information about these methods. Governments integrated these strategies into national public health agendas, ensuring their availability and accessibility.

## Polio eradication

Another success achieved through multi-stakeholder collaboration in global public health has been the eradication of polio. Global partnerships involving governments, international organizations, non-governmental organizations (NGOs), and community health workers have played important roles in this effort. For instance, the Global Polio Eradication Initiative (GPEI) united stakeholders across sectors to implement vaccination campaigns, surveillance systems, and community engagement strategies worldwide^20,21^. The impact of these coordinated efforts is significant. As of October 2023, there are only two endemic countries, Pakistan and Afghanistan, compared to an estimated 350 000 cases in over 125 endemic countries in 1988, a decrease of over 99% in wild poliovirus cases^22^. The collaborative efforts have brought the world closer to eradicating polio.

## Integration of digital health technologies in malaria control

In recent years, digital health technologies have revolutionized strategies for combating diseases and promoting public health^23,24^. Technology companies, public health agencies, researchers, and local communities have collaborated to develop innovations addressing various aspects of the malaria response, including diagnosis, management, microplanning, surveillance, and prevention.

A recent systematic review highlights several key technologies developed for malaria control worldwide. For example, a GIS-based spatial decision support system (SDSS) created by a team at the University of Queensland is used to quickly identify foci of active transmission, map the distribution of confirmed malaria cases, and direct focused interventions in elimination zones. In India, a Mobile-based Surveillance Quest using IT (MoSQuIT) automates and streamlines malaria surveillance, for all stakeholders from rural health workers to medical officers and public health decision-makers. Anti-malaria drones have also been widely used to spray biological insecticides in rice fields and swamps, reducing emerging mosquito populations in Kenya, Tanzania, India, Rwanda, and Zanzibar^25^.

These digital innovations and collaborations have significantly enhanced the effectiveness and efficiency of malaria control efforts.

## Opportunities and challenges

The perspectives brought forth by each stakeholder play an important role in promoting innovation and gaining an understanding of issues inherent in developing “made in Africa” solutions^26^. Moreover, the inclusion of emerging trends and innovations, such as digital health technologies, data analytics, global health security, and telemedicine platforms, is helping to reshape the landscape of collaborative governance in public health. These technological advancements can enable stakeholders to collaborate more easily, utilize real-time data for evidence-based decision-making, and facilitate remote engagement in policy development and implementation^27^. The utilization of digital tools also streamlines resource allocation and enhances the execution of initiatives.

Despite the evident benefits of collaboration, building partnerships presents its own set of challenges. A primary barrier is financial constraints. For instance, Sub-Saharan Africa currently has the lowest levels of government health spending per capita in the world^28^, which restricts the ability to invest in necessary resources, technologies, collaborations and training.

Differing priorities among stakeholders also pose a challenge, creating conflicts and slowing down decision-making processes. Additionally, power dynamics present another obstacle, this can lead to unequal participation and influence among stakeholders. Conflicting interests can also result in disagreements and fragmentation, further undermining the potential for cohesive action. However, by embracing a culture of trust, transparency, and shared purpose, these challenges can be reframed as opportunities for growth and innovation^29^.

## Recommendations for enhancing multi-stakeholder collaboration

Based on the identified opportunities and challenges, the following recommendations are proposed to enhance multi-stakeholder collaboration in public health:

## Strengthening multi-stakeholder platforms

Achieving excellent health and well-being extends beyond the capabilities of the health sector alone, it requires active social engagement, national awareness, and collaborations^30^. To realize this, governments must prioritize the development of integrated policy frameworks for aligning public health objectives across various sectors. Additionally, it is important to formalize multi-stakeholder platforms, as these can facilitate regular dialogue, coordination, and collaboration among diverse sectors.

## Developing innovative and sustainable funding models

Health financing solutions are not a ‘one size fits all’ approach; instead, they require tailored, country-specific strategies that address the unique needs of each country^31^. However, governments and international organizations can play an important role in developing and strengthening public-private partnerships, grants, and international funding mechanisms dedicated to collaborative public health initiatives^32,33^.

## Leveraging technology and data

In the digital era, capitalizing on and promoting the use of digital health technologies such as GIS-based systems, mobile surveillance platforms, and drones is important. These technologies have demonstrated the potential to enhance the effectiveness of public health interventions^25^. Furthermore, utilizing data analytics and real-time data for evidence-based decision-making can help to improve the accuracy of public health responses and facilitate timely interventions^24^. Expanding the use of telemedicine platforms to facilitate remote healthcare delivery and stakeholder engagement can also enhance access to healthcare services and ensure continuous collaboration, particularly in rural and underserved areas^34^.

## Promoting community engagement

Central to effective public health governance is the adherence to the Alma-Ata principle of community participation^14^. This principle underscores the importance of integrating the insights and experiences of grassroots organizations, community leaders, and marginalized individuals into decision-making processes, thereby facilitating the development and implementation of public health strategies that are relevant and culturally acceptable^35^.

## Conclusion

Attaining impactful and inclusive public health initiatives requires breaking down systematic barriers while creating progressive collaborations. The involvement of stakeholders across sectors is key in developing health initiatives and policies that truly transform lives. Importantly, integrating digital health technologies into public health initiatives can significantly enhance their effectiveness and reach. This approach can lead to positive change, reduce health inequalities, and achieve consonant health policies that benefit African society.

## Ethical approval

Not applicable.

## Consent

Not applicable.

## Source of funding

Not applicable.

## Author contribution

C.N.: conceptualization, data curation, investigation, writing—original draft, writing—review and editing, validation, and reviewing. W.K.B.P.: writing—original draft, writing—review and editing, validation, and reviewing. S.L.: writing—review and editing, and reviewing. M.S.M.: writing—review and editing, and reviewing. R.C.: writing—review and editing, and reviewing. W.N.: writing—review and editing, and reviewing. A.M.: writing—review and editing, and reviewing. All authors participated in reviewing, reading, and endorsing the final version of the paper.

## Conflicts of interest disclosure

C.N. is a consultant to Knowledge Action Change (K.A.C.). K.A.C. is a public health organization with a focus on harm reduction and is funded with a grant from Global Action to End Smoking (formerly known as Foundation for Smoke-Free World), an independent, U.S. nonprofit 501(c)(3) grantmaking organization. The views and opinions expressed in the contents of this paper are the sole responsibility of the authors, and should not, under any circumstances, be regarded as reflecting the positions of K.A.C. The remaining authors declare no conflicts of interest.

## Research registration unique identifying number (UIN)

All sources used have been duly cited.

## Guarantor

Chimwemwe Ngoma.

## Data availability statement

All sources used in the paper have been duly cited.

## Provenance and peer review

Not applicable.
